# Thermal buffering in a sessile insect herbivore

**DOI:** 10.1242/jeb.252214

**Published:** 2026-08-13

**Authors:** Austin R. Cruz, Judith L. Bronstein, Goggy Davidowitz

**Affiliations:** ^1^ Department of Ecology & Evolutionary Biology, 1041 E. Lowell Street, The University of Arizona, Tucson, AZ 85721, USA; ^2^ Department of Entomology, 1140 E. South Campus Drive, The University of Arizona, Tucson, AZ 85721, USA

**Keywords:** Scale insects, *Dactylopius*, Cochineal, Wax, Warming, Ecophysiology, Thermal ecology

## Abstract

For ectotherms such as insects, which have high surface area-to-volume ratios and are dependent upon environmental temperatures, physiological traits and/or a combination of behavioral mechanisms allow them to temporarily avoid sub-optimal or extreme temperatures typically found in hot, arid regions. However, sessile insects, such as cochineal (*Dactylopius* spp.) in the Sonoran Desert of the USA, are faced with the challenge of being minimally able to move to avoid extreme heat. Using artificial warming experiments and *in situ* thermography, we investigated the thermal function of the cottony wax produced over their bodies. We found that the wax buffers the cochineal from high temperatures relative to their host, and that the surface area of the wax reduces the temperature that the insects experience. These results reveal a mechanism for mitigating environmental exposure, a possible trade-off in defensive functions, and the importance of research on insect strategies for a warming climate.

## Teaser

**Summary:** Naturally produced wax covering the bodies of sessile scale insects (cochineal) reduces the temperature they experience on their host plants. The larger the surface area of wax, the greater the reduction in temperature.

## INTRODUCTION

All organisms must regulate their responses to the environment in order to grow, survive and reproduce. Temperature is a dominant and ubiquitous component of the environment for many species because it governs key organismal processes that span biological levels of organization, from physiological (Gillooly et al., 2001) to population-level dynamics (Savage et al., 2004). Insects are especially sensitive to the thermal environment as ectotherms, owing to high surface-area-to-volume ratios (Kühsel et al., 2017) that make them vulnerable to thermal stress and desiccation. The diverse repertoire of strategies that insects have evolved to avoid these challenges (e.g. behavioral, such as movement, and physiological, such as the development of a waxy cuticle) is well documented (see González-Tokman et al., 2020). Here, we investigated a less-studied mechanism by which sessile insects mitigate exposure to high temperatures. A better understanding of the ecological strategies that diverse taxa employ in response to elevated temperature is critical, as it can enhance our predictive abilities of their persistence in a rapidly warming world.

Cochineal (*Dactylopius* spp.) are scale insects (Hemiptera) native to arid, predominantly hot, regions of the Americas. Currently, there are 11 recognized species in the monogeneric family Dactylopiidae, with six species endemic to North America and five species endemic to South America (García Morales et al., 2016). Juvenile females obligately feed on various species of Cactaceae, especially *Opuntia* spp., and permanently settle in one location on their host after inserting their stylets into the cactus tissue for nutrients. Once settled, females mature into wingless adults and continue to feed. Notably, females often aggregate in large clusters on their host cactus. The adult males, unlike the females, are mobile, winged, short-lived and do not develop mouthparts, and therefore cannot feed (Pérez Guerra, 1991; Schowalter, 2025). Culturally, cochineal insects are well known. For centuries, these insects (specifically, *D. coccus*) have been domesticated and harvested by indigenous communities throughout the Americas for the use of a scarlet dye produced from carminic acid in the insect's hemolymph and acquired from their dried, crushed bodies (Donkin, 1977; Pérez Guerra, 1991; Schowalter, 2025). The word cochineal is derived from the Greek and refers specifically to this color. Biologically, carminic acid is used as a defense against the cochineal insects' primary predators in the study region, including a larval and adult beetle (*Hyperaspis trifurcata*), a moth larva (*Laetilia coccidivora*) and a fly larva (*Leucopis bellula*) (Eisner et al., 1980; Gilreath and Smith, 1988; Eisner et al., 1994; Vanegas-Rico et al., 2010; Kelly et al., 2022).

**Fig. 1.**
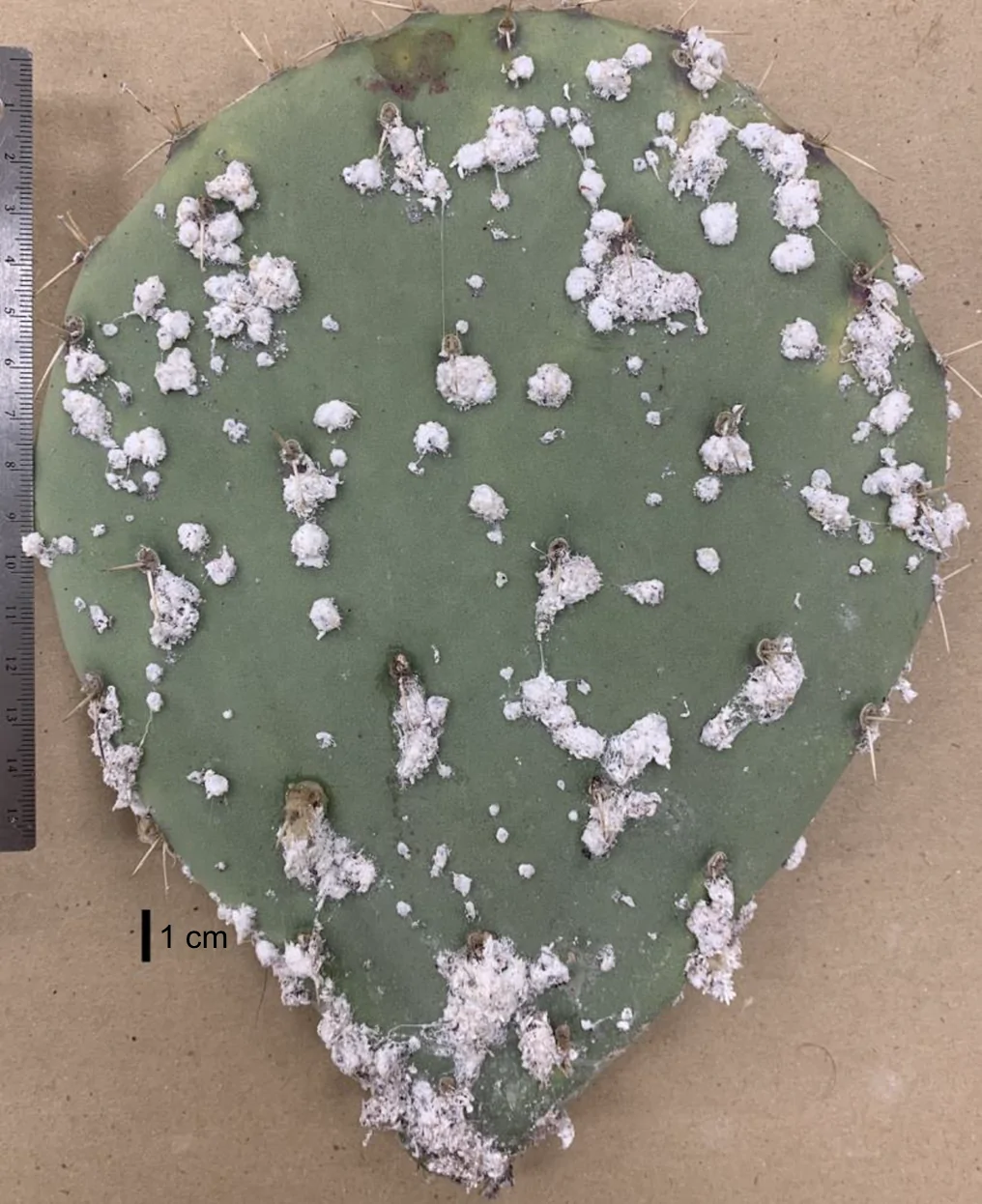
**A single cladode removed from host cactus (*Opuntia engelmannii*) with cochineal (*Dactylopius* sp.) clusters.** Note: scale bar and ruler (in centimeters).

In contrast to the extensive research on the function and uses of carminic acid, the cochineal insects' production of conspicuous, white, cottony, cuticular wax (Fig. 1) has received limited scientific attention (Meinwald et al., 1975; Foldi, 1991; Gullan and Kosztarab, 1997). The wax is secreted from glands under the base of their setae and forms dense, thread-like structures (filaments); males typically produce shorter wax filaments than females, and only on their abdominal segments, whereas females produce longer filaments on their heads, thorax and abdomen that cover the whole body (Pérez Guerra, 1991; Schowalter, 2025). The wax has been shown to offer minimal protection against predators and parasites (Moran, 1980; Cruz-Rodríguez et al., 2016; Kelly et al., 2022; A.R.C., personal observation). There is little to no current knowledge of how it might alter abiotic environmental effects on the cochineal (Moran and Hoffmann, 1987). Given the sessile life history of female adults, any moderation of the hot, arid conditions on the cactus pad should be highly beneficial.

Here, we investigated a possible thermoregulatory function of cuticular wax production in cochineal insects. We hypothesized that the wax buffers the insects from high temperatures (hypothesis 1). We predicted that (a) cochineal insects with wax that has been experimentally removed would be warmer than the surface of the cactus pad upon which they are feeding (because they are darker than the cactus pad), and (b) individuals covered by wax would be cooler relative to the surface of the cactus pad than those with wax that has been removed. The null prediction was that the temperature of cochineal insects would be the same as the surface of cactus pad regardless of treatment. Next, we hypothesized that the surface area of the wax affects the temperature that the insects experience (hypothesis 2). We predicted that the larger the surface area of wax (i.e. the larger the cluster of females), the greater the reduction in temperature that the insects experience relative to the cactus pad because an increased surface area of wax provides a larger surface to reflect solar radiation. The null prediction was that the change in temperature would be independent from the size in wax surface area.

## MATERIALS AND METHODS

### Field collections

Two *Opuntia engelmannii* (hereafter, ‘cactus’) cladodes (‘pads’) with abundant cochineal were haphazardly selected and harvested (Fig. 1) from a large individual cactus (approximately 1 m height, 2 m diameter) on the University of Arizona (UA) campus in Tucson, AZ, USA (32°14′55.7304″N, 110°59′14.7084″W), on 23 November 2020 and maintained at ambient laboratory conditions. The harvested pads were separated with a blade by cutting the joint that connects the bottom of the pad with another pad on the same individual plant. Although we were unable to identify the species of cochineal owing to difficulty in distinguishing morphological traits (but see Pérez Guerra, 1991), the two native species found in the local area are *Dactylopius opuntiae* and *Dactylopius confusus*, the latter often associated with its host *O. engelmannii* (Kelly et al., 2022).

In a separate observational period, we used a thermal camera (FLIR T-300, Wilsonville, OR, USA) to collect *in situ* infrared images of *Dactylopius* sp. on several individual *O. engelmannii* to record possible deviations in temperature between cochineal clusters and pad surfaces. The imaging was conducted within 0.5 m of the cactus in the aforementioned location of the university campus on 29 January 2020 (at approximately 14:30 h) and on 19 February 2020 (at approximately 15:00 h) for approximately 15 min on each date. All thermography data were recorded in degrees Celsius (°C). Historic air temperature, collected by the Department of Hydrology and Atmospheric Sciences at the UA Physics and Atmospheric Sciences Building (32°13′47.5″N, 110°57′14.3″W), approximately 245 m from the location of the cactus, on 29 January 2020 at 14:30 h was 23.8°C with a relative humidity (RH) of 40%, and on 19 February 2020 at 15:00 h was 22.9°C with a RH of 40% (www.atmo.arizona.edu/index.php?section=weather). Thermal camera settings for emissivity (ε) and RH were 0.98 and 50%, respectively. Complete camera metadata were extracted using the flirsettings function in the ‘Thermimage’ package (v. 4.1.3; https://CRAN.R-project.org/package=Thermimage) in R software (v. 4.2.1; https://www.r-project.org/), and are available as a separate file from the Zenodo repository (see doi:10.5281/zenodo.20788494).

### Experiment 1: effect of wax removal (hypothesis 1)

In a laboratory setting, six cochineal insect clusters (multiple individual females aggregated together naturally) of various surface area sizes were haphazardly selected from one cactus pad on 27 November 2020. To avoid disturbing the insects and the natural coverage of the wax, we did not count the number of females present beneath the wax. The cactus pad was first experimentally warmed with an infrared (IR) bulb (wavelength range of 700–2400 nm with a peak around 1190 nm; R40 250 W, General Electric Company, Cleveland, OH, USA) placed 71 cm directly above the pad, at an angle of approximately 90 deg relative to the pad, for 5 min. A pilot study (unpublished) confirmed that this height and duration consistently reproduced an ecologically relevant temperature range (between 28°C and 38°C, reflecting monthly average high temperatures throughout most of the year at the study site) (www.weather.gov/twc/TucsonMonthlyNormalExtremes). The temperatures both beneath each cochineal cluster and outside and adjacent to the cluster were measured using micro-thermocouples (Omega type-T mini thermocouple HYPO-33-1-T-G-60-SMP-M with 0.2 mm diameter, and an Omega HH23A thermocouple reader that can record from two thermocouples simultaneously; Omega Engineering, Norwalk, CT, USA) and recorded, representing our control. The thermocouple outside the cluster was unshielded (but see Maclean et al., 2021) and placed within 1–3 mm of the cluster. Care was taken that it was nearly vertically positioned (pointing downwards) and in direct contact with the pad. We manually calculated the temperature differences between thermocouples, where 0°C indicates no difference in temperature beneath the cochineal cluster and the surface of the cactus pad. A positive value of °C indicates a higher temperature beneath the cluster than on the pad surface, and a negative value of °C indicates a lower temperature beneath the cluster than on the pad surface.

After the warming treatment, a period of approximately 30 min elapsed to allow the cochineal to re-equilibrate to ambient (baseline) temperature. The majority of wax covering each cochineal cluster was then gently removed with a soft paint brush to expose the individual bodies of each cochineal (see Fig. S1 for example of typical amount of wax removed from each cluster). This process minimally disturbed the insects, as it did not dislodge them from the pad but only removed the wax from their bodies. The exposed cochineal clusters were then experimentally warmed with the IR bulb as above (the bulb was placed 71 cm above the pad for 5 min). The temperatures beneath each cochineal cluster and outside and adjacent to the cluster were again measured with micro-thermocouples and recorded over a period of approximately 5–7 min, representing our ‘treatment’. Again, we manually calculated the temperature differences between each thermocouple.

### Experiment 2: effect of wax area (hypothesis 2)

In a laboratory setting, 15 cochineal clusters were haphazardly selected from one of two sides of the two individual cactus pads. Another set of 15 cochineal clusters was selected from the same side of an individual pad, yielding a total of 75 unique clusters (15 clusters/side of cactus pad ×5 pad sides). Photographs were taken of each side of the two individual cactus pads and the surface area of all unique cochineal clusters was measured using ImageJ software (Schneider et al., 2012) and reported in units of cm^2^. First, the temperatures both beneath each cochineal cluster and outside and adjacent to the cluster were measured using micro-thermocouples (described above) and recorded (control). The thermocouple outside the cluster was unshielded and placed within 1–3 mm of the cluster, and care was taken that it was nearly vertically positioned (pointing downwards) and in direct contact with the pad. Next, the same side of the cactus pad surface was experimentally warmed with the IR bulb (as described above), and the temperatures both beneath each cochineal cluster and outside and adjacent to the cluster were measured and recorded in a spreadsheet (warming treatment). All temperature differences were manually calculated between each thermocouple in both the control and warming treatments. This entire process was repeated five times between 23 and 25 November 2020, yielding a total of 75 paired control and warming treatment observations, with each treatment lasting approximately 10 min to collect and record temperatures.

### Analysis

All statistical analyses were conducted in R (v. 4.2.1). To assess the effect of the natural presence of wax on the temperature of cochineal clusters (experiment 1), we conducted a Wilcoxon signed-rank test using the ‘wilcox.test’ function in the ‘stats’ package (R v. 4.2.1) to examine the mean differences between the control (with wax) and the treatment (with wax removed) and calculated the effect size (*r*) using the ‘wilcox_effsize’ function in the ‘rstatix’ package (v. 0.7.2; https://CRAN.R-project.org/package=rstatix). To assess the effect of wax surface area on the temperature of cochineal clusters (experiment 2), the surface area values were log transformed and an ordinary least squares (OLS) regression was performed using the ‘smatr’ package (v. 3.4.8; Warton et al., 2012) for both control and warming treatments. We reported the slope (α) and elevation (β) regression coefficients for each treatment group as well as the Pearson correlation coefficient using the ‘cor.test’ function in the ‘stats’ package. Results were visualized with the ‘ggplot2’ package (v. 3.5.1; Wickham, 2016).

## RESULTS AND DISCUSSION

### *In situ* thermography

**Fig. 2.**
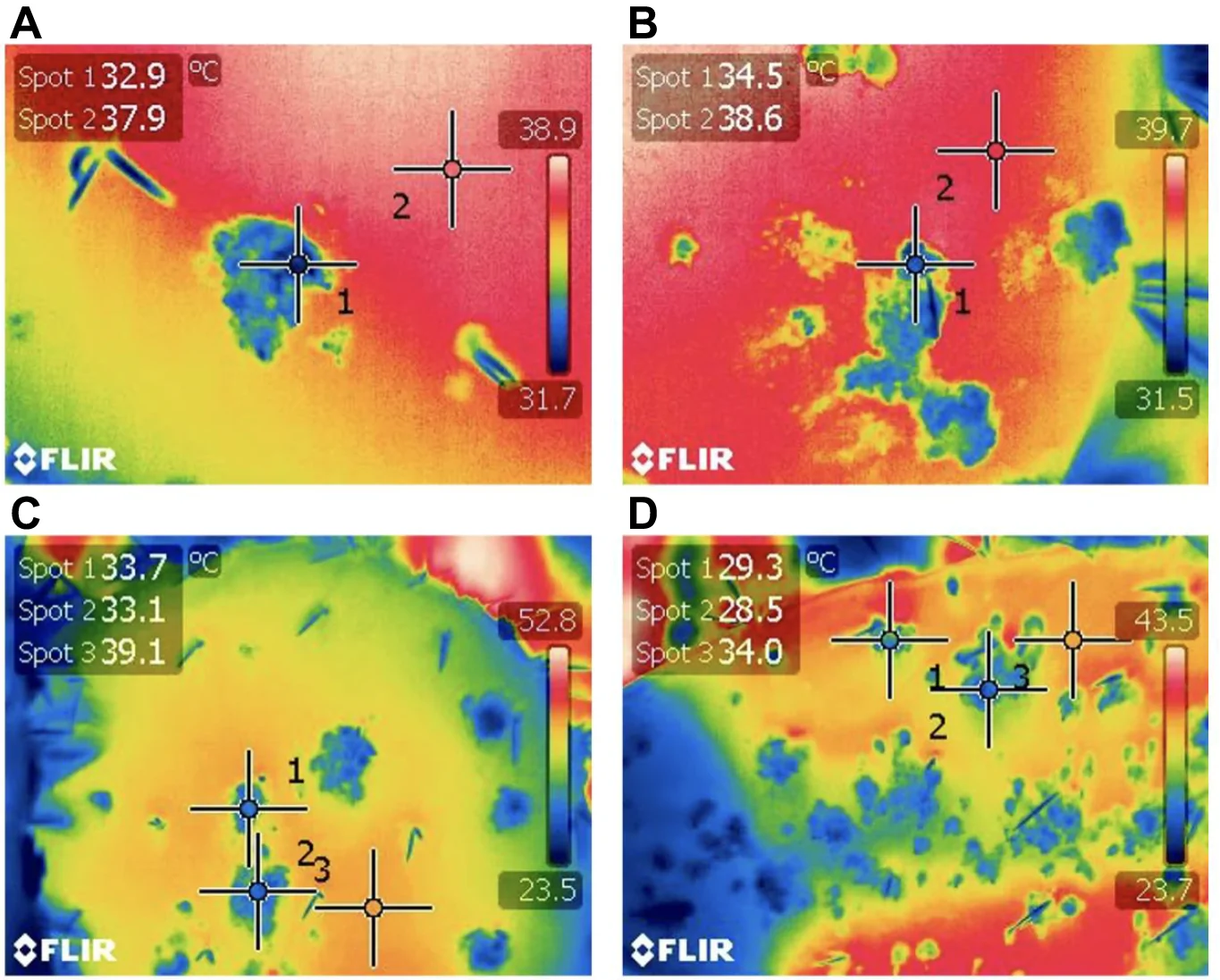
***In situ* thermography of cochineal on host cactus.** In each panel, the cochineal clusters are colored blue, indicating lower relative temperatures (°C), whereas the surface of the cactus pad is predominantly yellow or red, indicating higher relative temperatures (see temperature scale inset in each panel). Each panel represents a different individual cactus pad.

The *in situ* thermography of cochineal clusters revealed that cochineal insects on cactus pads showed large thermal differences (∼4–6°C cooler) relative to the surface tissue of their host (Fig. 2). The emergent pattern from these thermal images is the presence of relatively cooler, blue ‘islands’ of cochineal insect clusters surrounded by relatively hot, red cactus surface area. Descriptive statistics of the thermography and complete absolute temperature data are available in Table S1.

### Experiment 1: wax removal

**Fig. 3.**
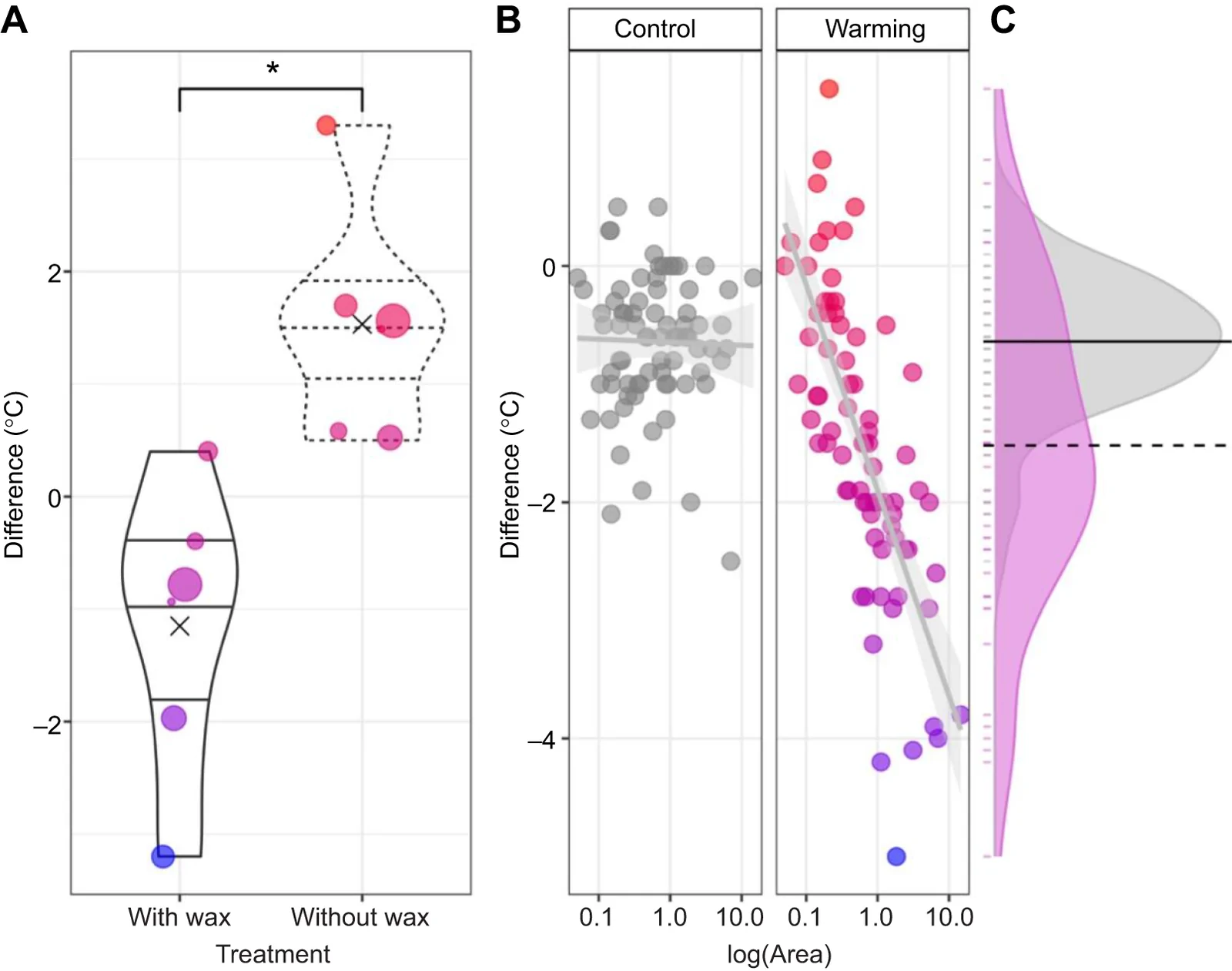
**The effect of wax on thermal buffering of cochineal insects across experimental treatments.** (A) Violin plots of two cochineal treatments (with wax, *n*=6; without wax, *n*=6) and their thermal responses to increasing temperatures of the cactus pad. Violin plots illustrate 0%, 25%, 50%, 75% and 100% percentiles, color indicates temperature (blue, low; red, high), and circle size indicates size of the cochineal individual or cluster. Mean temperature difference values are illustrated by ×. (B) The effect of wax area on cochineal thermal responses to increasing temperatures of the cactus pad, grouped by category (control, *n*=75; warming, *n*=75). Colored circles in the warming treatment indicate difference in temperature relative to the caucus pad surface (blue, low; red, high), and regression lines are reported with their respective 95% confidence intervals (gray shaded bands). Note: 0°C indicates no difference in temperature beneath the cochineal cluster and the cactus pad. (C) Density plots of response variable distribution for B. Gray distribution (mean, solid line) corresponds to the control treatment, pink distribution (mean, dashed line) corresponds to the warming treatment, and short colored horizontal lines left of the density plots indicate the distribution of specific data points in B.

Cochineal insect clusters (range=0.05–14.668 cm^2^, average=1.35, median=0.618, s.d.=2.14) with wax were significantly cooler than those with wax that had been experimentally removed (*Z*_5_=−2.15, *P*=0.03, *r=*0.90; Fig. 3A). We observed a median temperature difference of −0.85°C (mean±s.d.=−1.15±1.27°C) between the cochineal clusters with wax and the surface of the pad, and a median temperature difference of 1.55°C (mean±s.d.=1.53±1.01°C) between the cochineal clusters without wax and the surface of the pad, for an absolute median difference of 2.4°C between the two treatments. Descriptive statistics and complete absolute temperature data for experiment 1 are available in Table S2 and as a separate file (see doi:10.5281/zenodo.20788494).

### Experiment 2: wax surface area

The greater the surface area of wax produced by an individual or cluster of individuals, the larger the reduction in ambient temperature measured for the cochineal insects compared with an adjacent area of the cactus pad surface in the warming treatment (α*=*−1.74, 95% CI=−2.12, −1.36, β=−1.90, 95% CI=−2.12, −1.68, *R^2^*=0.54, *P*<0.001; Fig. 3B; Fig. S1) versus the control (α*=*−0.03, 95% CI=−0.27, 0.22, β=−0.64, 95% CI=−0.79, −0.50, *R^2^*=0.0006, *P*=0.83), which had a slope indistinguishable from 0 (Fig. 3B; Fig. S2). A significant negative correlation between a reduction in temperature and wax surface area was observed in the warming treatment (*R*=−0.73, 95% CI=−0.82, −0.61, *P*<0.001) but not in the control (*R*=−0.025, 95% CI=−0.25, 0.20, *P=*0.83). Descriptive statistics and complete absolute temperature data for experiment 2 are available in Table S3 and as a separate file (see doi:10.5281/zenodo.20788494).

Cochineal scale insects are well known for their carminic acid and its biotic effects on natural enemies (Pérez Guerra, 1991; Schowalter, 2025). In contrast to the attention paid to this trait, related in part to its significant cultural importance (Donkin, 1977; Schowalter, 2025), the function of this insect's conspicuous cuticular wax has received surprisingly little scientific attention. Our experiments suggest that the wax of Sonoran Desert *Dactylopius* helps reduce temperatures relative to the surface of the pad of their host cactus (*Opuntia engelmannii*) (Fig. 3A). Further, the larger the surface area of the secreted wax, the more moderated the temperature that the insects underneath experience relative to the cactus, providing in some instances ∼4–6°C difference in thermal buffering (Fig. 3B). This result is also consistent with the thermal reduction observed *in situ* (Fig. 2). Our work is the first to investigate a novel, thermally protective function of the cochineal wax in response to the thermal environment.

The production of protective wax by other insect species has also been shown to mediate the abiotic effects that these organisms experience. For example, also in the Sonoran Desert, tenebrionid beetles (*Cryptoglossa verrucosa, Eleodes armada*) secrete a cuticular wax that may change their reflectance, thereby reducing heat load and affecting thermoregulation and evaporative water loss (Hadley, 1970, 1979). Similar functional properties have also been proposed for various species of tenebrionid beetle in the Namib Desert that differentially produce cuticular wax along a humidity gradient (McClain et al., 1985). For sessile cochineal insects, cuticular wax likely regulates evaporative water loss, which is critical for insects in arid regions and those with high surface area-to-volume ratios (Brückner et al., 2017), while also reducing exposure to solar radiation.

Interestingly, the wax from *Dactylopius* has one of the highest melting points (∼100°C) among insect-produced waxes (Tulloch, 1970). A high melting point of wax would be crucial for withstanding extreme summer heat of the Sonoran Desert, the hottest of the four North American deserts (Weiss and Overpeck, 2005). For example, cladode temperatures of up to 50°C have been recorded in July (mid-summer) on *O. engelmannii* (Cockell et al., 2004). Furthermore, the white coloration of cochineal wax (Cloudsley-Thompson, 1979) might confer additional protection from other sources of electromagnetic radiation, such as UV (Beard, 1972), via decreased absorption (but see Wang et al., 2021). Our thermography (Fig. 2) data indicate that exposed cactus surfaces were 5.1°C above cochineal clusters (Table S1), suggesting that radiative energy is likely a strong selective pressure that the wax mitigates. Further research on the biophysical properties of the wax and the pad (e.g. their reflectance) can help clarify this function and the extent to which the observed thermal buffering enhances survival and/or reproduction (for discussion on fecundity, see Moran and Cobby, 1979). Until then, we emphasize a preliminary interpretation of our results given the absence of reflectance data for this system.

How metabolically costly such a wax is to produce remains an open question, but comparative evidence suggests that this cost might trade off with other defensive functions. For example, the species *D. coccus* has historically been domesticated; as a result, the insects are larger (∼2×) than other species in the genus and produce a fine waxy powder over their bodies (Pérez Guerra, 1991). This is distinctly different from the robust, cottony wax found in non-domesticated species (Donkin, 1977). This domesticated species, which invests less in a waxy covering, produces higher concentrations of carminic acid than does the non-domesticated *D. opuntiae* (Barreto-García et al., 2020). It is possible that wax production might trade off with carminic acid concentrations, which can have significant negative fitness effects on its primary predator *Laetilia coccidivora* (Barreto-García et al., 2020). This offers a compelling hypothesis about the physiological trade-off between abiotic and biotic defenses in this system.

Moving forward, research on the thermal ecology of cochineal might benefit by extending analyses across spatial and temporal scales as well as across *Dactylopius* species. For example, how might the micro- and macro-thermal context influence physiological investment into, and potential plasticity of, wax production? At the micro-thermal scale, it would be interesting to investigate whether more cochineal clusters are located on north-facing pads, or whether the clusters are bigger on the south-facing pads (Gibbs and Patten, 1970; Moran et al., 1982; Pérez Guerra, 1991). Additionally, research on whether cochineal remain within, or emerge from, the surface boundary layer of its host could provide insights into the role of convection as another cooling mechanism for scale insects (Pincebourde et al., 2021). Although we only investigated the protective effects of the wax at high temperatures, there might be additional benefits of the wax, such as cold hardiness (Sinclair et al., 2003), that are conferred at low or freezing temperatures, which can occur in the Sonoran Desert during the winter months (but see Weiss and Overpeck, 2005). In a heterogeneous macro-environment, large thermal gradients are often found across the landscape and can play an important role in the association of insects with host plants (Pincebourde et al., 2021). Moreover, how might diurnal and seasonal changes in temperature affect wax production and its protective effects? Finally, might thermal protection, which increases with the area of wax coverage (Fig. 3B), have selected for aggregation behavior in the sessile females? Addressing these questions will provide greater insights into the evolutionary ecology and phylogenetics of cochineal thermal physiology.

As a whole, our work uncovers a novel mechanism by which cochineal insects mitigate the effects of high temperatures associated with their host cactus. In addition to providing better insights into organismal thermal biology, our research raises questions about the broader integration between physiological and ecological responses of species in a warming world. The answers to these questions will be important as climate warming accelerates across the globe and species are increasingly exposed to thermal stress.

## Supplementary Material

10.1242/jexbio.252214_sup1 Supplementary information

## Data and resource availability

All data and analysis code are available from Zenodo (https://doi.org/10.5281/zenodo.20788494). All other relevant data and details of resources can be found within the article and its supplementary information.
