# Supplementary material for: Thermal buffering in a sessile insect herbivore
Source: J Exp Biol. 2026 Aug 13;229(16):jeb252214. doi: 10.1242/jeb.252214 (PMC13535094; doi:10.1242/jeb.252214)
Supplement: Supplementary file 1 [file jexbio-229-252214-s1.pdf]

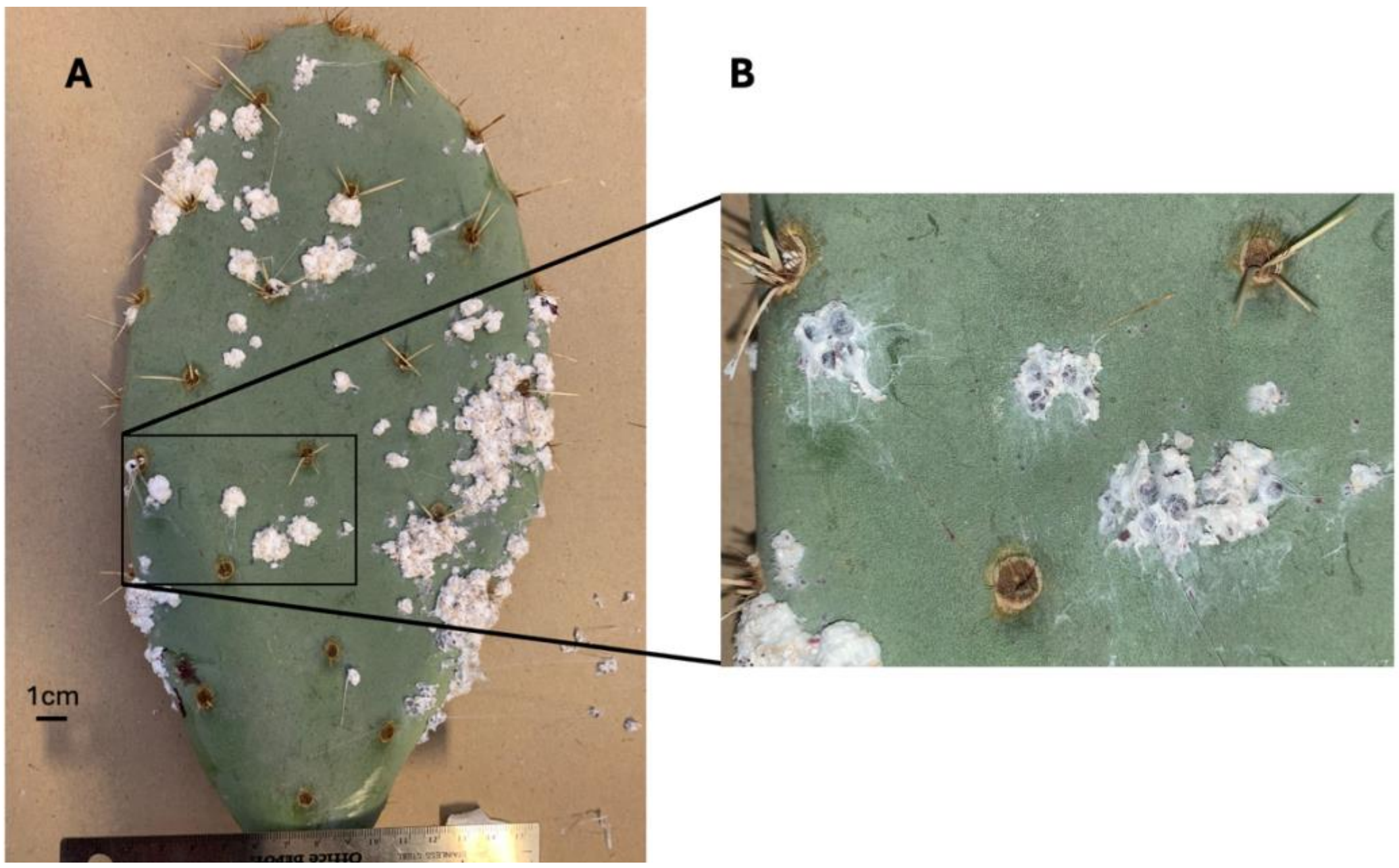

Fig. S1. An example of typical amount of wax removal in Experiment 1. (A) Cactus pad with boxed outline illustrating cochineal clusters in the control (no wax removed). (B) Cochineal clusters from boxed inset with wax removed (treatment). Note: 1cm scale bar (left) and ruler (bottom) in (A).

Journal of Experimental Biology • Supplementary information

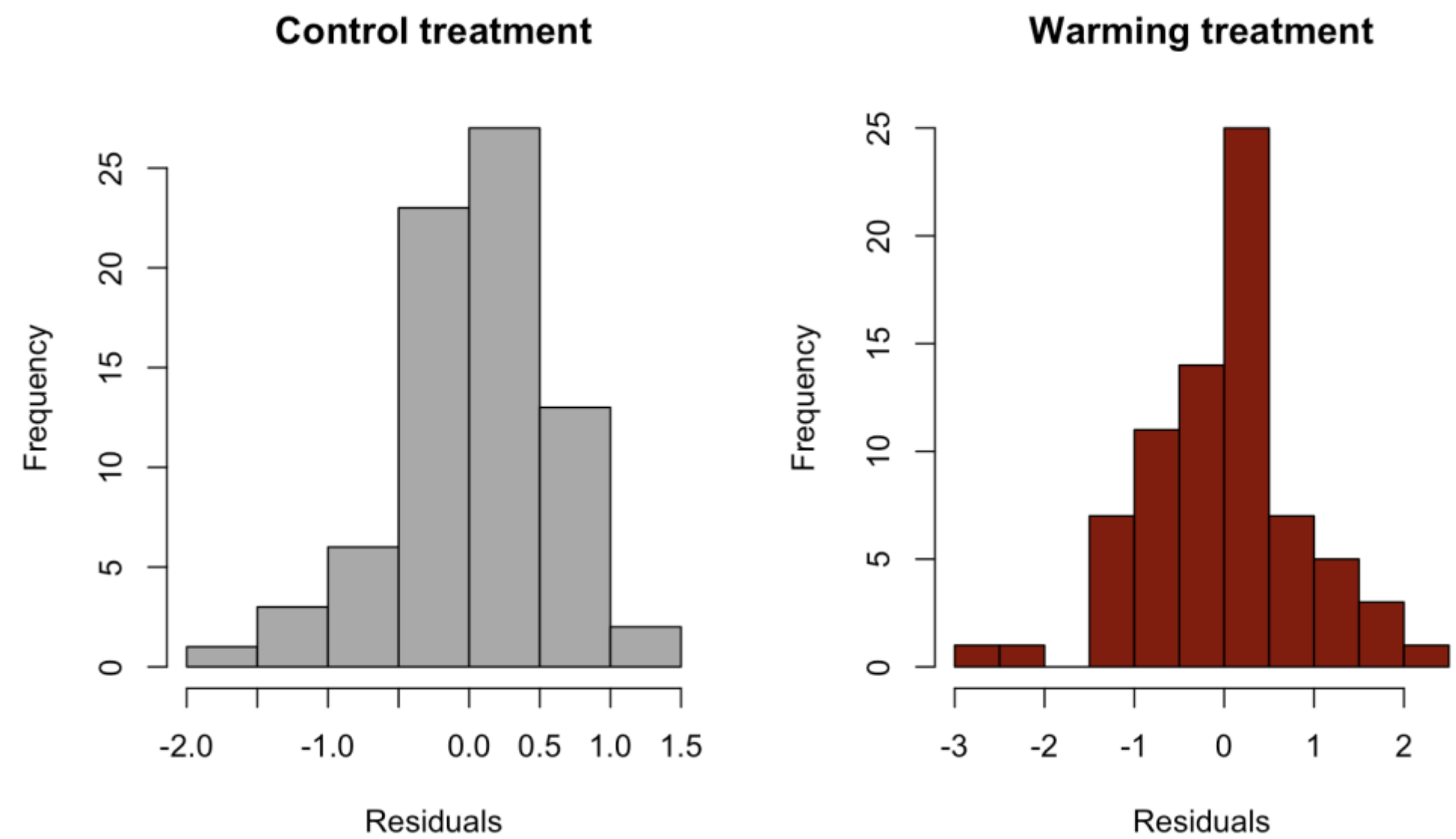

Fig. S2. Residual distributions of differences in temperature (response variable) for each treatment in experiment #2.

Table S1. Temperature measurements in thermal images in Figure 2 (main text). Note that the reported cochineal cluster temperatures are averages of the two measurements in panels C and D.

Descriptive statistic	Panel	Air temp (°C)	Cochineal cluster (°C)	Cactus pad surface (°C)	Sample size (*n*) of measured cochineal cluster temperatures
	A	22.9	32.9	37.9	1
	B	22.9	34.5	38.6	1
	C	23.8	33.4	39.1	2
	D	23.8	28.9	34	2
Mean		23.35	32.43	37.4	
Standard deviation		0.52	2.44	2.32	
Median		23.35	33.15	38.25	

Table S2. Descriptive statistics for experiment 1.

	Warming with wax		Warming without wax	
**Descriptive statistic**	**Cactus pad surface (°C) (*n* = 6)**	**Cochineal cluster (°C) (*n* = 6)**	**Cactus pad surface (°C) (*n* = 6)**	**Cochineal cluster (°C) (*n* = 6)**
Min.	30.2	28.2	29.4	29.9
Max.	35.4	34.6	35.1	36.8
Mean	32.86667	31.71667	31.96667	33.5
Standard deviation	2.0973	2.268406	2.281812	2.841127
Median	33.4	32	31.5	33.5

Table S3. Descriptive statistics for each cactus pad side (5 total) used in control and warming treatment for experiment 2.

		**Control**		**Warming**	
**Cactus # / side #**	**Descriptive statistic**	**Cactus pad surface (°C) (*n* = 15)**	**Cochineal cluster (°C) (*n* = 15)**	**Cactus pad surface (°C) (*n* = 15)**	**Cochineal cluster (°C) (*n* = 15)**
1/1	Min	21	19.9	28	24.1
	Max	23.1	22.6	36.6	36.2
	Mean	22.17333	21.63333	32.79333	30.78667
	Standard deviation	0.667048	0.896554	3.191387	3.64101
	Median	22.2	21.6	34.4	31.3
1/2	Min	19.5	18.7	25.3	24.5
	Max	22.2	21.3	37.6	35.7
	Mean	20.68667	19.90667	30.07333	28.22
	Standard deviation	0.883068	0.85228	3.759078	3.305882
	Median	20.7	19.8	30.4	27.6
2/1	Min	17.7	16.6	25.4	23.2
	Max	20.8	20.5	35	34
	Mean	18.98667	18.20667	28.4	27.06
	Standard deviation	1.001332	1.029193	2.823625	3.529225
	Median	19	18.1	26.8	25.1
2/2	Min	24.5	24.1	31	28.3
	Max	25.8	25.5	41.5	39.6
	Mean	25.30667	25.02	35.11333	33.27333
	Standard deviation	0.39545	0.402137	3.7175	3.925278
	Median	25.4	25	33.9	32.6
1/2	Min	18.8	18.1	24.5	23
	Max	21.9	21.3	34.3	33.7
	Mean	20.31333	19.50667	29.24667	28.68667
	Standard deviation	1.009856	0.991728	2.896517	3.039235
	Median	20.2	19.5	29.5	29

Journal of Experimental Biology • Supplementary information
